# Hybrid Time–Frequency Analysis for Micromobility-Based Indirect Bridge Health Monitoring

**DOI:** 10.3390/s25247482

**Published:** 2025-12-09

**Authors:** Premjeet Singh, Harsha Agarwal, Ayan Sadhu

**Affiliations:** Department of Civil and Environmental Engineering, Western University, London, ON N6A 5B9, Canada; psing225@uwo.ca (P.S.); hagarwa6@uwo.ca (H.A.)

**Keywords:** indirect bridge health monitoring, micro-mobility devices, modal identification, frequency analysis, damping ratio, wavelet packet transform, synchro-extracting transform

## Abstract

Bridges serve as vital connectors in the transportation network and infrastructure. Given their significance, it is crucial to continuously monitor bridge conditions to ensure the efficient operation of transportation systems. With advancements in sensing technologies, transportation infrastructure assessment has evolved through the integration of structural health monitoring (SHM) methodologies. Traditionally, bridge monitoring has relied on direct sensor instrumentation; however, this method encounters practical obstacles, including traffic disruptions and limited sensor availability. In contrast, indirect bridge health monitoring (iBHM) utilizes data from moving traffic on the bridge itself. This innovative approach eliminates the need for embedded instrumentation, as sensors on vehicles traverse the bridge, capturing the dynamic characteristics of the bridge. In this paper, system identification methods are explored to analyze the acceleration data gathered using a bicycle-mounted sensor traversing the bridge. To explore the feasibility of this micromobility-based approach, bridge responses are measured under varying traversing conditions combined with dynamic rider–bicycle–bridge interaction for comprehensive evaluation. The proposed method involves a hybrid approach combining Wavelet Packet Transform (WPT) with Synchro-extracting Transform (SET), which are employed to analyze the time–frequency characteristics of the acceleration signals of bike-based iBHM. The results indicate that the combination of WPT-SET demonstrates superior robustness and accuracy in isolating dominant nonstationary frequencies. The performance of the proposed method is compared with another prominent signal processing algorithm known as Time-Varying Filtering Empirical Mode Decomposition (TVF-EMD). Ultimately, this study underscores the potential of bicycles as low-cost, mobile sensing platforms for iBHM that are otherwise inaccessible to motorized vehicles.

## 1. Introduction

Civil infrastructure is fundamental in supporting the economic development of a country, with bridges serving as a critical transportation link for the movement of goods and people. With the rising volume of traffic and freight size, bridges are subjected to more demanding loading conditions than originally designed for. According to the Infrastructure Report Card by the American Society of Civil Engineers (ASCE), of all the bridges, 42% are at least 50 years old and 7.5% are classified as structurally deficient. Aging, sustained traffic loads, and environmental exposure result in progressive deterioration that needs to be regularly monitored to ensure structural integrity. Traditionally, bridge monitoring was based on visual inspection and periodic testing campaigns to assess the physical and functional classification of the bridge [1]. However, these methods are limited in their ability to detect subsurface damage and are subject to inspector’s subjectivity. To overcome the limitations of the traditional monitoring approaches, recent advancements in bridge health monitoring (BHM) have enabled continuous surveillance and facilitated early detection of structural defects.

Vibration-based BHM is a widely adopted technique through which structural integrity is assessed using the dynamic response of the bridge [2,3]. The fundamental principle of this approach is that structural damage leads to an alteration in physical properties such as stiffness, mass, or damping, which will be reflected as a measurable change in dynamic properties. This approach constitutes an inverse problem, where observed dynamic behavior is used to estimate the internal condition of the structure. Implementation typically involves the direct instrumentation of sensors, such as accelerometers, at strategically chosen locations on the bridge, commonly referred to as direct BHM. A critical component of direct BHM is the careful selection and placement of sensors to accurately capture the critical physical parameters [4]. Thus, structural degradation can be traced by monitoring changes in natural frequencies, mode shapes, and damping properties inherently related to stiffness, mass, and damping [5].

BHM methodologies are further categorized based on parameters such as inspection scale, response type, system behavior, and computational framework, highlighting both model-based and data-driven paradigms [6]. Despite its effectiveness, the major drawback of conventional BHM involves high installation costs, lane or road closures, the need for traffic management, and mounting limitations, which are strongly influenced by the typology and number of sensors deployed [7]. To address these limitations, a vehicle-induced vibration strategy—referred to as drive-by or indirect bridge health monitoring (iBHM)—has emerged as a promising alternative [8].

iBHM enables effective bridge excitation through regular traffic flow, reducing noise interference and eliminating the need for costly and intrusive instrumentation. This approach has further minimized the dependency on fixed sensors, cables, and acquisition systems by extracting damage-sensitive features directly from vehicle-mounted sensors. This principle was initially explored by [9] and later validated through numerical studies and experiments on simply supported beams [10]. More recently, advancements in iBHM have explored vibration-based approaches using moving vehicles with varied exciter–receiver configurations ranging from specialized monitoring vehicles [10] to passenger cars and trucks [11]. [12] reviewed and categorized the iBHM techniques based on the damage characteristics being analyzed. These categories included (i) methods based on natural frequencies, (ii) mode shapes, (iii) curvature or strain mode shape-based approaches, and (iv) hybrid methods that utilize the combination of modal parameters.

Recent studies have introduced scalable, low-cost, and minimally disruptive monitoring solutions using lightweight vehicles [13,14,15] such as bikes and scooters for iBHM applications. One such framework [16] utilized smartphone sensors temporarily mounted on shared micromobility vehicles to monitor an in-service footbridge using crowd-sourced data. Subsequently, work by [17] expanded upon this concept through field experiments on a bicycle and a pedestrian bridge. Simultaneous monitoring of the vehicle and bridge reduced ambiguity in the results, and the use of pedaling cadence was proposed to enhance the visibility of the bridge’s dynamic modes. These findings underscore the potential of low-speed and low-mass vehicles as sustainable and cost-effective solutions, making them ideal for monitoring sensitive or inaccessible structures, such as footbridges [18].

Despite significant advancements by various researchers, a persistent challenge in iBHM remains consistent acquisition and effective analysis of dynamic vehicle response. The time-varying nature of vehicle bridge interaction (VBI) resulting from noisy vehicle response due to a combination of factors such as vehicle speed, vehicle suspension, surface roughness, limited sensor measurement, operational conditions, etc., has received limited attention in the literature. This data is analyzed using time-domain, frequency-domain, and time-frequency methods to monitor changes in structural parameters over time [19]. Utilizing robust signal processing techniques is crucial for indicating changes in the vibration response of structures, which is essential in the processes of damage localization, quantification, and detection [20].

Many vibration-based damage assessment methods rely on modal properties from the Fourier transform (FT). However, as a data reduction process, it may lose crucial information about the structural condition [21]. It also struggles to capture evolving signal characteristics and often misses localized damage represented by closely spaced, poorly excited higher frequency modes, which makes FT-based damage identification challenging [22]. Using an expectation-maximization algorithm, a Bayesian approach was developed by [23]. The proposed method could compute the most probable value of modal parameters. A comparison with three existing algorithms revealed that the proposed method identified global mode shape accurately; however, it was challenged by the low-quality data.

In another vein, Ref. [24] developed a structural reliability analysis using a quantified, active-learning Kriging-based method. The methodology was based on an updated probability density function, which was created using weights based on an improved learning function. The method featured a probabilistic-based stopping criterion for the training phase. In the past two decades, TF methods have gained popularity, but most require multi-channel measurements, making them unsuitable for system identification (SID) with fewer sensors [25]. To address these challenges in a unified fashion, a Wavelet-based TF method has been employed for its decomposition and denoising capabilities using a fewer number of sensors.

In this paper, Wavelet Packet Transform (WPT) has been explored to track the bridge dynamic behavior for improved modal parameter identification. It simplifies damage detection by using a single-channel nonstationary measurement, significantly reducing user input while boosting accuracy and reliability in structural assessments. For instance, Ref. [22] introduced a statistical SHM method based on wavelet packet decomposition (WPD) and statistical process control (SPC). Further, in a study by [26], a vibration-based SHM method using (WPT) and Karhunen–Loève transform for pattern recognition is deployed. It detects changes in a composite beam’s behavior, achieving up to 95% classification accuracy, and shows greater sensitivity to variations than traditional Fourier analysis, proving its effectiveness for damage-sensitive pattern recognition in SHM.

WPT is well-suited to analyzing nonstationary signals with multiple embedded frequency components. It serves as a preprocessor, separating the analyzed signal into a set of narrow-band signals [27]. Hence, it is more robust and less susceptible to changing working conditions. Ref. [28] utilized WPT component energy and FEM updating to detect structural damage. They accurately identified damage in noisy conditions, outperforming the discrete wavelet transform energy, though this method may produce false peaks in heavy noise. In this study, WPT is used as a decomposition tool to split the acceleration signal into its mono-components. As the signal originating from VBI is time-varying in nature, the resulting WPT coefficients do not provide adequate TF information related to the time-varying nature of the signal. Therefore, WPT has been integrated with another powerful TF method known as Synchro-Extracting Transform (SET). SET has been developed to improve the energy concentration of TF representations by retaining only the data related to time-varying features of the parent signal [29]. It is well-suited to analyze nonstationary signals with multiple embedded frequency components.

To evaluate the effectiveness of the proposed method, comprising WPT and SET, a hybrid SID method has been included in this study. Empirical Mode Decomposition (EMD) is a technique used to break down a multi-component signal into its simpler components, which are called intrinsic mode functions (IMFs) [25,30]. Recent studies have shown that data-driven signal decomposition methods such as Variational Mode Decomposition (VMD) can outperform classical empirical approaches for SHM tasks due to their superior mode separation and noise robustness [31]. While EMD is adaptable, Ref. [32] presented how Time-Varying Filter-based EMD (TVF-EMD) addresses the limitations of traditional EMD in isolating time-varying frequencies and mitigating mode mixing. It utilizes a flexible time-varying filter for the sifting process, enhancing instantaneous amplitude and frequency estimation [33]. Overall, TVF-EMD is adaptive and effective for analyzing linear and nonstationary signals, offering better frequency separation and stability compared to traditional EMD [34]. Due to these qualities, TVF-EMD has been selected for a direct comparison with the WPT to undertake iBHM using a micromobility device (bicycle). This study proposes a sparse SID method based on WPT and SET and provides a comparison with an established TF method, TVF-EMD.

Earlier iBHM works primarily focused on feasibility demonstrations and data collection frameworks using bicycles. In contrast, this study advances the field by introducing a comparative methodological evaluation of WPT–SET against TVF–EMD. Furthermore, while WPT and SET have been applied separately in SHM, to the best of the authors’ knowledge, their integration has not previously been explored in micromobility-based iBHM contexts. The paper is structured as follows: after introducing the concept of iBHM and the associated challenges, the proposed method is briefly introduced in this section. Section 2 provides the background of WPT, SET and TVF-EMD, and formulation of the proposed method. Section 3 presents a description of the bridge, experimental details, and data used in this study. Section 4 provides a discussion on the results of the proposed method, followed by key conclusions in Section 5.

## 2. Proposed Methodology

This study presents a comparative signal processing framework for analyzing acceleration data collected by sensors instrumented on the bridge and the bicycle. WPT and TVF-EMD are examined for feature extraction and modal identification in conjunction with the SET.

WPT offers a complete decomposition of both low- and high-frequency components, providing better frequency resolution across the spectrum. Wavelet thresholding is applied using soft or hard shrinkage rules to remove noise prior to decomposition. The denoised time-domain acceleration signal measured from either the bridge-mounted or bicycle-mounted sensor, *x*(*t*), is recursively decomposed into $2^{j}$ sub-bands at level j using orthonormal wavelet packet bases, $\psi_{j,k}(t)$:

(1) $$x\left(t\right)=\sum_{j=1}^{J}\sum_{k=1}^{2^{j}}c_{j,k}\left(t\right)\cdot \psi_{j,k}\left(t\right)$$

where $c_{j,k}(t)$ are the wavelet packet coefficients. SET is applied to selected wavelet coefficients to analyze energy concentration and frequency modulations.

(2) $$S_{j,k}\left(t,\omega \right)=\int c_{j,k}\left(u\right)h\left(t-u\right)e^{-jwu}du$$

where h(t) is the window function localized around time t and $e^{-jwu}$ is the complex exponential used to extract localized frequency content. Contrary to classical wavelet transforms, SET improves the localization of signal components by adaptively tracking instantaneous frequency (IF) ridges. WPT decomposition levels are identified that correspond to the expected modal frequency bands based on preliminary frequency-domain analysis of the data. The energy distribution within these bands is evaluated to select coefficients that capture dominant vibration responses and verify consistency across multiple trials to exclude coefficients influenced primarily by noise. Features such as dominant frequency and damping ratio are extracted from the SET output for selected coefficients. The dominant frequency $f_{d}(t)$ can be defined as the frequency with the maximum energy concentration in the SET TF spectrum.

The dominant frequency for Equation (2) is

(3) $$f_{d}\left(t\right)={\arg max}_{\omega }\left|S_{j,k}(t,\omega )\right|$$

To extract the damping ratio from the SET output, the logarithmic decay method with exponential curve fitting is utilized.

(4) $$A\left(t\right)=A_{0}e^{-\zeta \omega_{n}t}$$

From Equation (2), we obtain

(5) $$A\left(t\right)=\left|S_{j,k}\left(t,\omega =2\pi f_{d}\left(t\right)\right)\right|$$

From Equations (4) and (5), we obtain

(6) $$\zeta \left(t\right)=-\frac{1}{\omega_{d}\left(t\right)}\cdot \frac{d}{dt}\left[\ln\left|S_{j,k}\left(t,\omega_{d}\left(t\right)\right)\right|\right]$$

To evaluate the performance of the WPT-SET approach, another signal processing method is used in this study. TVF-EMD is an improved EMD variant that features adaptive filtering to extract intrinsic mode functions (IMFs) with reduced model mixing and enhanced frequency localization. The raw acceleration signal x(t) is decomposed into a finite number of IMFs using adaptive bandpass filters:

(7) $$x\left(t\right)=\;\sum_{k=1}^{K}{IMF}_{k}(t)+\;r\left(t\right)$$

where ${IMF}_{k}(t)$ represents the k-th model and r(t) is the residual. SET is applied to selected IMFs to extract a high-resolution TF representation. For the k-th IMF obtained from TVF-EMD, SET is applied as

(8) $$S_{{IMF}_{k}}\left(t,\omega \right)=\int {IMF}_{k}\left(u\right)\cdot h\left(t-u\right)\cdot e^{-jwu}du$$

Modal parameters such as dominant frequency and damping ratio are determined from the SET from Equation (4).

(9) $$f_{d}\left(t\right)={\arg max}_{\omega }\left|S_{{IMF}_{k}}(t,\omega )\right|$$

(10) $$\;\;\;\zeta \left(t\right)=-\frac{1}{\omega_{d}\left(t\right)}\cdot \frac{d}{dt}\left[\ln\left|S_{{IMF}_{k}}\left(t,\omega_{d}\left(t\right)\right)\right|\right]$$

**Figure 1:**
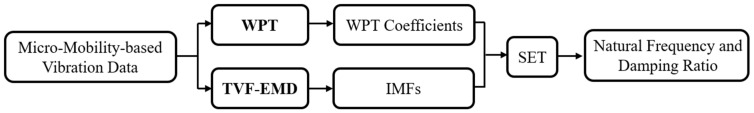
Flowchart of the proposed methodology.

The comparative methodology featured in this study is shown in Figure 1. To compare the efficacy of the proposed signal processing methods, both WPT and TVF-EMD are applied to the acceleration sensor data collected through various pedaling scenarios outlined in the next section. Time-series acceleration signals are obtained from sensors instrumented on the bridge and a traversing bicycle. The dynamic response of the bridge under moving loads is captured in the acceleration signal, which is used as an input for comparative analysis. The output from WPT and TVF-EMD is used as an input for the SET algorithm, which provides TF representation of the coefficients from WPT and IMFs from TVF-EMD. The modal parameters extracted from SET are compared across different sensor positions, including mid-span and quarter-span, to determine the bridge’s natural frequencies and damping ratio.

## 3. Experimental Setup and Data Collection

### 3.1. Bridge Description

**Figure 2:**
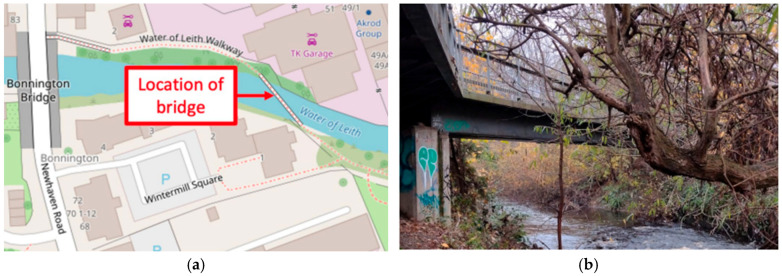
Pedestrian bridge: (**a**) location and (**b**) photograph [35].

The study was conducted on a 30-meter-span pedestrian and bicycle bridge by [35], as shown in Figure 2, which is assumed to behave as a simply supported beam. While controlled and ambient excitation methods were used to investigate the bridge’s modal properties, accelerometer sensors were installed at multiple key locations along the span, including the mid-span and quarter-span points, enabling a comprehensive modal analysis. The accelerometer sensors were magnetically attached to the bridge through a non-structural surface layer.

### 3.2. Data Description

The dataset used in the study was acquired through a series of field experiments conducted in two distinct phases: (i) baseline SID under ambient and pedestrian heel-drop excitation and (ii) bicycle traversals featuring varying rider input dynamics. For the proposed research, emphasis is placed on Phase (ii) data, which is well-suited for iBHM via bicycle-mounted sensors. Each phase was designed to capture the bridge’s dynamic response under different excitation scenarios and environmental conditions.

**Figure 3:**
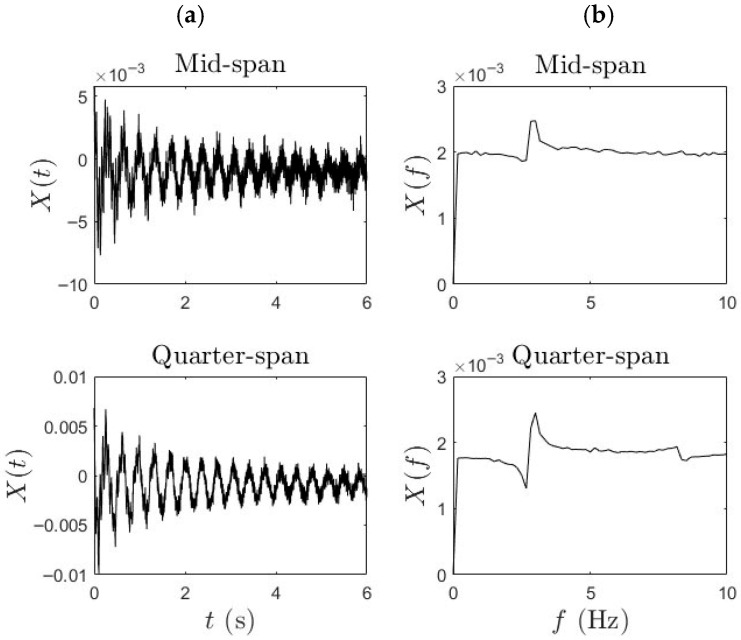
(**a**) Time history and (**b**) FFT of the typical bridge response.

Phase (i) data was important to establish a baseline reference for the bridge’s modal properties. In this phase, controlled heel-drop tests were performed by pedestrians at various key points along the span, particularly at the mid-span and quarter-span. These heel drops served as near-impulse excitations and were separated by sufficient intervals (~10 s) to ensure that the bridge’s dynamic response was not affected by residual vibrations from previous excitations. To obtain the spatially representative dynamic behavior of the bridge, wireless MEMS accelerometers with a sampling rate of 512 Hz using LORD Micro Strain G-Link-200 devices were mounted at mid-span and quarter-span locations on the bridge deck, while an additional sensor was installed centrally on the bicycle frame. This configuration enabled the simultaneous acquisition of synchronized acceleration data from both the bridge and the moving bicycle, thereby facilitating the indirect identification of bridge modal properties. The typical acceleration data and its frequency domain representation for Phase (i) are shown below in Figure 3. Figure 3a presents the measured acceleration responses in the time domain at the mid-span and quarter-span of the bridge, while Figure 3b shows their corresponding frequency-domain representations obtained using the Fast Fourier Transform (FFT). The FFT plots highlight distinct spectral peaks, which correspond to the bridge’s modal frequencies, evident in both measurement locations. These frequency peaks serve as key indicators for identifying the structural vibration characteristics and evaluating damping properties.

**Figure 4:**
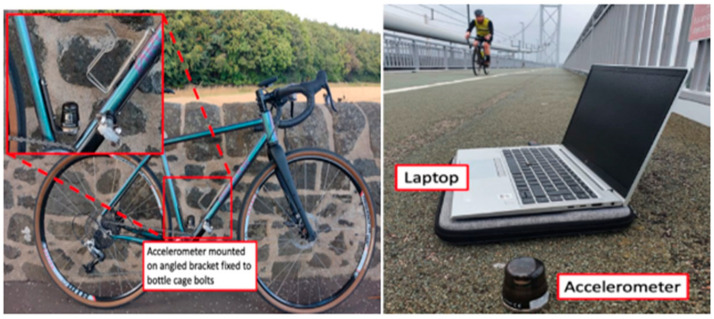
Data gathering and acquisition process [35].

The data collection in Phase (ii) was aimed at evaluating the feasibility of bicycle-based iBHM. Figure 4 depicts the data acquisition process using the sensors instrumented on the bicycle and the bridge surface. The testing involved multiple bicycle passes across the bridge under different operational conditions to simulate varying rider-induced dynamic inputs. The interaction of the rider–bicycle–bridge system was analyzed under three conditions—freewheeling, slow cadence, and fast cadence—capturing the effects of harmonic input forces introduced by pedaling. Freewheeling traversal comprises no pedaling force, therefore allowing natural rider–bicycle dynamics to excite the structure. Slow pedaling trials use sprocket 5 (38 × 19 gear ratio), while fast pedaling consists of trials using sprocket 9 (38 × 32 gear ratio) to induce higher-frequency harmonic input [35].

By picking frequency peaks in the bicycle-mounted sensor’s acceleration response, corresponding to the bicycle passing over the deck joints as it entered and exited the bridge, span traversal speed was determined. Traversal speeds ranged between 3 m/s and 5 m/s, with each traversal generating an individual data sample lasting approximately 7.2 s. Sensors were placed both on the bridge deck and on the bicycle to enable direct and indirect modal parameter estimation. The same sensor type was used for both the bridge and the bicycle, ensuring uniformity in signal characteristics and simplifying post-processing. All sensor data were sampled at 512 Hz, and no artificial filtering was applied beyond the device’s built-in 1.5 kHz anti-aliasing filter. The wireless transmission of calibrated signals also minimized the need for external signal conditioning, streamlining data acquisition and analysis.

**Figure 5:**
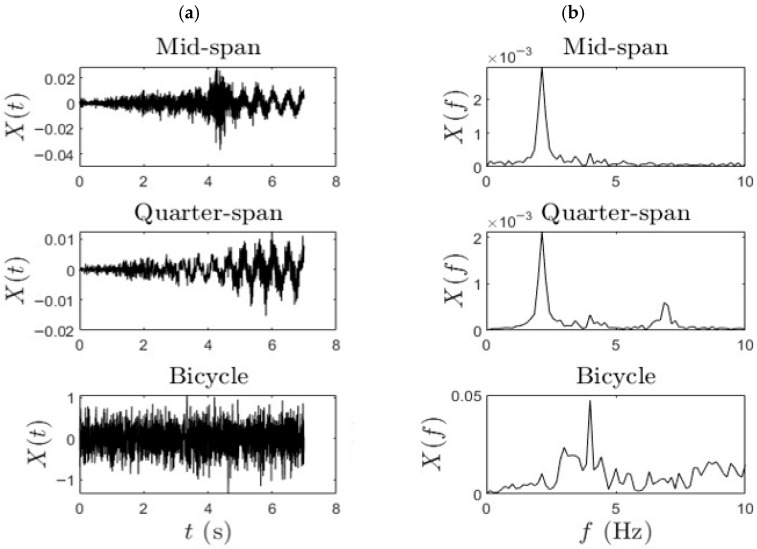
(**a**) Time history and (**b**) FFT of the typical bridge and bicycle response.

The typical acceleration data and the corresponding frequency-domain representation obtained via the Fast Fourier Transform (FFT) of the signal for Phase (ii) data are represented in Figure 5. These plots were derived from acceleration signals recorded during controlled bicycle traversals over the bridge. This data was further analyzed to extract the modal frequency content and damping ratio associated with the bridge structure. In the FFT plot, the observed bridge frequency peak (2.14 Hz) corresponds to the modal frequency of the bridge evident in the mid-span and quarter-span plots.

## 4. Results

In this section, the results generated using the TF methods, as discussed in Section 2, are demonstrated. The data from the bicycle sensor and bridge sensors at quarter-span and mid-span locations are analyzed using WPT and SET. The frequencies and damping ratio values are identified using WPT and SET. Similar results are shown for the TVF-EMD and TVF-EMD-SET frameworks, and a comparison of the two alternatives is provided.

### 4.1. WPT-SET

**Figure 6:**
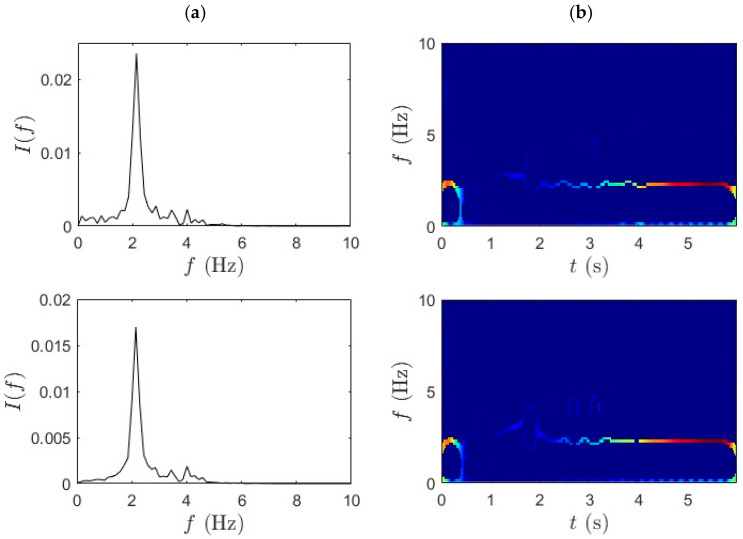

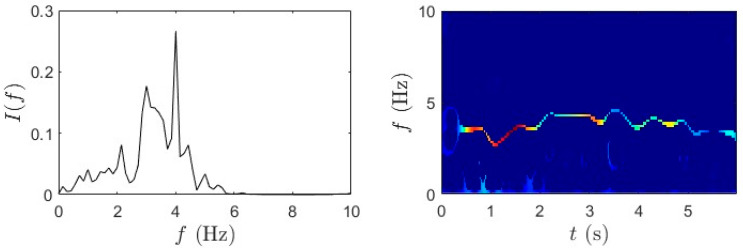
(**a**) WPT and (**b**) SET results for slow pedaling trial 1 for bridge mid-span, bridge quarter-span, and bicycle.

**Figure 7:**
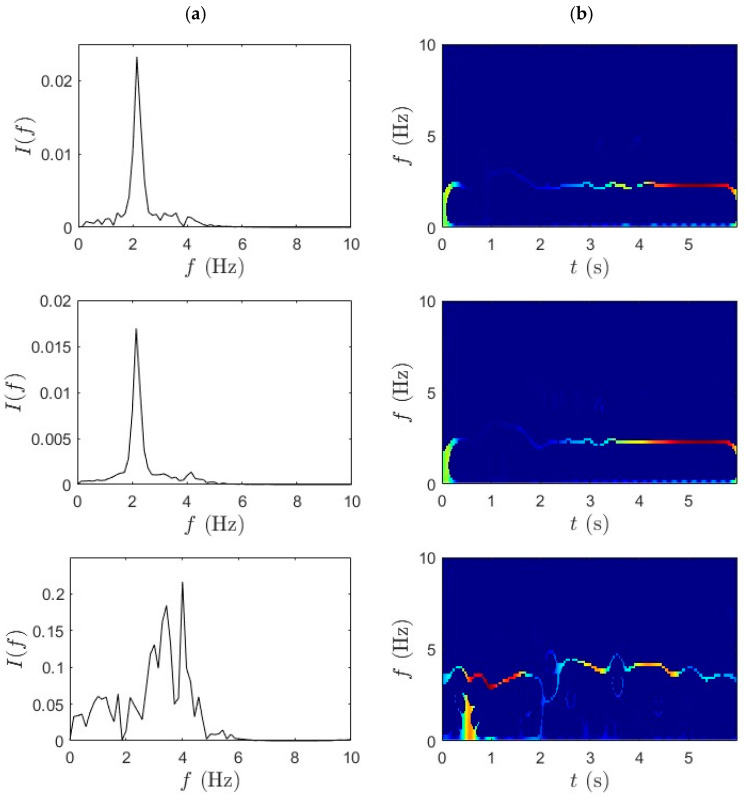
(**a**) WPT and (**b**) SET results for slow pedaling trial 2 for bridge mid-span, bridge quarter-span, and bicycle.

This section employs the proposed method to analyze the acceleration data collected from the bridge-mounted and bicycle-mounted sensors. The WPT decomposition provides a structured and hierarchically resolved frequency band separation, which facilitates precise identification of modal frequencies. The window length is set to 256 samples (0.5 s) at a 512 Hz sampling rate, providing a balanced trade-off between time and frequency resolution for the bridge’s fundamental modes (~2–3 Hz). The SET frequency grid is defined with a resolution of 0.02 Hz, sufficient to capture subtle modal frequency variations while maintaining computational efficiency. Local maxima of the SET TF energy distribution are first identified, and continuous IF trajectories with the highest cumulative energy are selected as dominant ridges. These ridges are then used to compute the corresponding damping ratios through exponential curve fitting of amplitude decay. Figure 6 and Figure 7 show the frequency identification results for slow pedaling trials 1 and 2, respectively. Column (a) in each figure corresponds to the WPT results, while column (b) illustrates the SET-based outcomes. The data is segmented based on sensor location to capture spatial variability. The first row represents measurements at the bridge mid-span, the second row corresponds to the quarter-span location, and the third row shows the bicycle-mounted accelerometer sensor response. This segmentation enables direct comparison between structural responses across the span and vehicle-induced vibrations transmitted through the bridge system.

For bridge-based sensors in the first two rows, the WPT isolates primary bridge modes with reduced mode mixing and enhanced spectral contrast. This outcome is particularly important for addressing the challenge of separating closely spaced modal frequencies in nonstationary signals—a key limitation in iBHM. The decomposition results show sharply defined peaks in the spectral domain, demonstrating WPT’s capability to capture modal responses under operational traffic conditions. The bicycle-mounted data from the third row also reveals consistent frequency bands related to vehicle dynamics.

This finding reinforces the suitability of micromobility-induced vibrations as a viable excitation source for iBHM. The SET results, shown in Figure 6b and Figure 7b, complement the WPT decomposition by providing a highly concentrated and temporally localized frequency ridge. In the current analysis, SET highlights well-localized frequency ridges with minimal cross-component interference, especially in the mid-span and quarter-span data. The IF trajectories remain consistent and continuous over time, demonstrating the stability of modal features under slow pedaling excitation.

**Figure 8:**
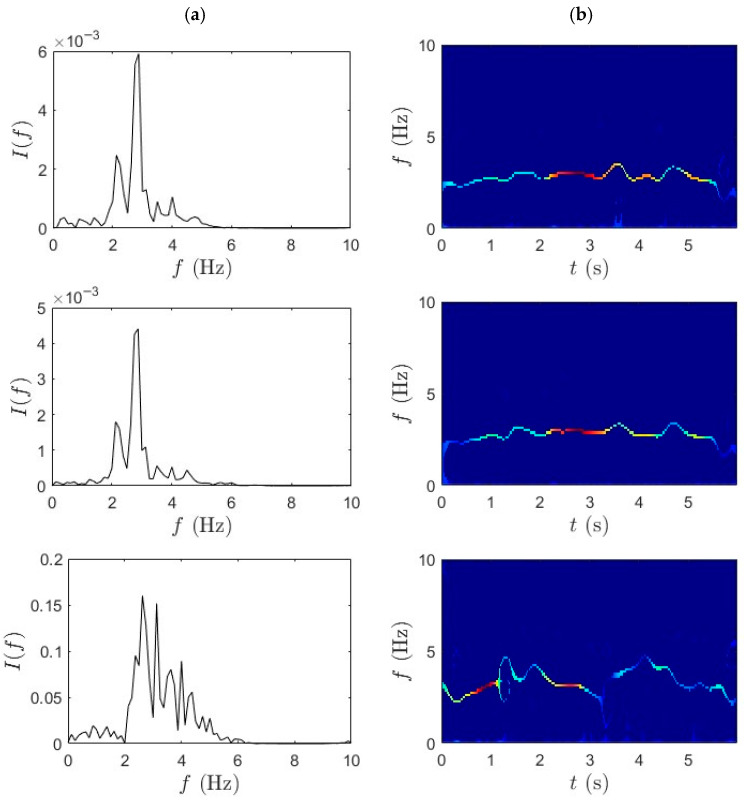
(**a**) WPT and (**b**) SET results for fast pedaling trial 1 for bridge mid-span, bridge quarter-span, and bicycle.

**Figure 9:**
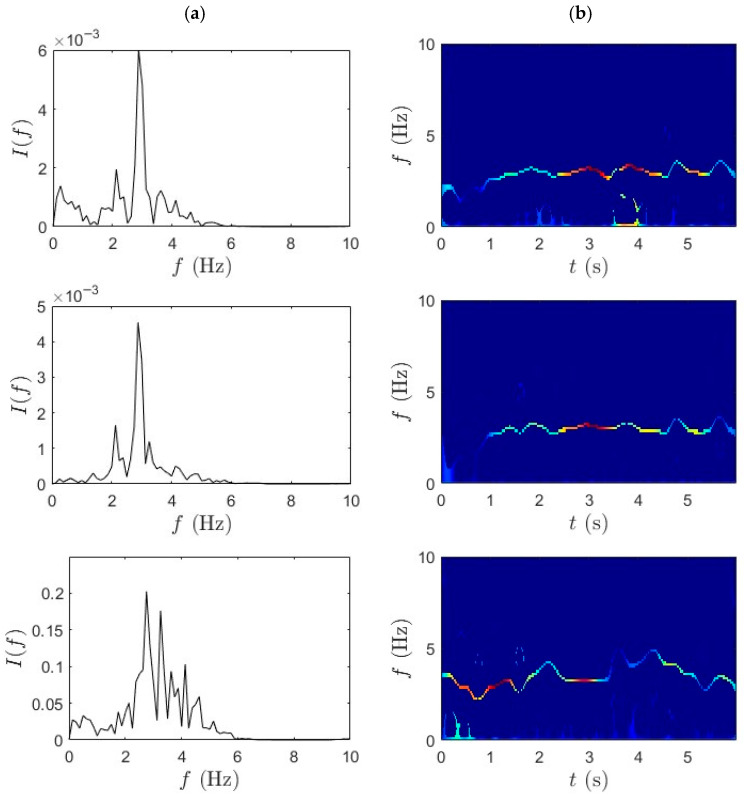
(**a**) WPT and (**b**) SET results for fast pedaling trial 2 for bridge mid-span, bridge quarter-span, and bicycle.

Similarly, Figure 8 and Figure 9 provide the TF characterization of acceleration signals collected during fast pedaling trials 1 and 2, analyzed using the WPT followed by the SET. The consistent layout and segmentation of rows and columns is maintained to have a transparent comparison across test conditions. Even under intensified signals generated by fast pedaling, WPT demonstrates robust decomposition capability, effectively separating multiple active frequency components within the shortened traversal window. Clear frequency band boundaries are maintained even under the more complex vibratory conditions induced by higher cycling speeds. The mid-span signal predominantly reveals the lower bridge modes, while the quarter-span signal focuses on slightly elevated modal frequencies. The bicycle response signifies a broader frequency range, reflective of enhanced bridge–bicycle coupling at increased velocity. Importantly, despite the denser spectral content and reduced observation time, WPT exhibits stable decomposition performance without pronounced mode interference.

Figure 8b and Figure 9b presents the outcome of applying SET to WPT-decomposed data. The integration of SET yields a more refined TF distribution characterized by sharply defined IF ridges, with high energy concentration and minimal spectral drift. Across all three sensor locations, SET successfully captures transient modal shifts with precision, mapping the dominant frequencies without excessive dispersion. The bicycle-mounted sensor further benefits from this combined approach, producing spectral bands, with minimal overlap and strong definition. The synergistic application of WPT and SET facilitates accurate extraction of modal parameters, including natural frequencies and damping ratio.

Table 1 summarizes the dominant frequencies and corresponding damping ratios identified using the WPT and SET across various sensor locations during both slow and fast pedaling trials. During slow pedaling, both the mid-span and quarter-span sensors consistently detected a structural frequency of approximately 2.14 Hz, which was observed across multiple trials. This consistency indicates the robustness of the WPT-SET framework in isolating bridge modal frequencies with high precision, even under relatively low excitation conditions. The bicycle-mounted sensor shows a steady detection at 4 Hz, corresponding to the inherent dynamics of the bicycle–rider system. However, in trial 3, a slight variation is observed in the bicycle frequency at 3.50 Hz, suggesting that irregular riding or the interaction between the bridge and bicycle may have contributed to slight variability in the measured response. Such deviations align with prior findings that vehicle-induced excitations are inherently sensitive to rider input and contact conditions [10]. The damping ratio values reduce from slow pedaling trials to fast pedaling trials; however, no clear trend could be identified, which can be attributed to differences in pedaling excitation, environmental influences, and inherent estimation uncertainties in operational modal analysis.

Under fast pedaling conditions, the identified modal characteristics exhibited notable shifts. The bridge frequency increased to 2.87 Hz in trials 1 and 2 and further to 3 Hz in trial 3. The bicycle frequencies also exhibit slight variation, with several closely spaced values such as 2.62 Hz, 2.75 Hz, and 2.87 Hz. This spread reflects the increased complexity and coupling effects experienced during high-speed traversal. The first natural frequency of the bridge, 2.85 Hz, identified in the benchmark dataset study [35] aligns closely with the results from the fast-pedaling conditions. Frequency values identified in the fast-pedaling trials using WPT and SET match closely with the benchmark frequency value identified using forced excitation [35]. Overall, the results demonstrate the robustness and accuracy of WPT in identifying modal frequencies under both mild and intense dynamic conditions. There is a strong consistency in the bridge response, while the bicycle data shows acceptable variability due to operational differences.

### 4.2. TVF-EMD-SET

**Figure 10:**
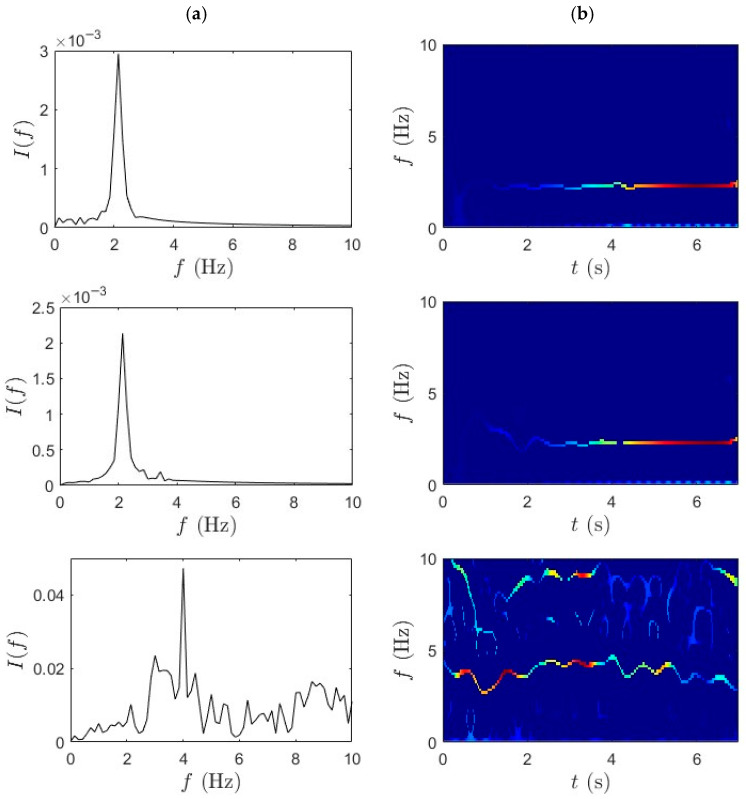
(**a**) TVF-EMD and (**b**) SET results for slow pedaling trial 1 for bridge mid-span, bridge quarter-span, and bicycle.

**Figure 11:**
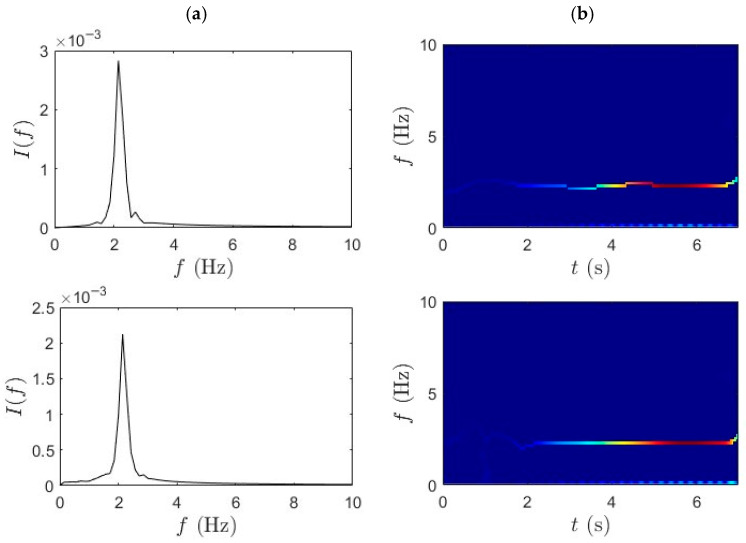

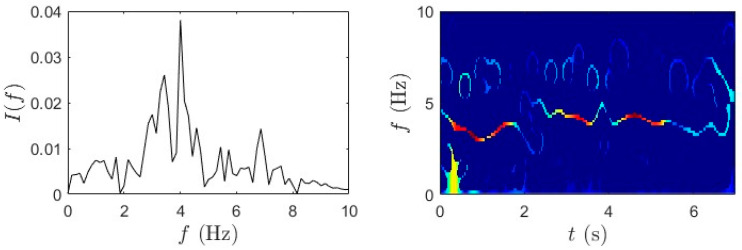
(**a**) TVF-EMD and (**b**) SET results for slow pedaling trial 2 for bridge mid-span, bridge quarter-span, and bicycle.

In this section, analysis using TVF-EMD and SET was carried out on the same dataset for performance comparison with the proposed method. The adaptive TVF bandwidth is set empirically to 0.2–0.3 Hz, allowing adequate separation of IMFs corresponding to the bridge’s modal frequencies (~2–3 Hz) while suppressing high-frequency noise components. The sifting process is terminated when the standard deviation between successive IMFs falls below 0.2% or after a maximum of 10 iterations per IMF. Figure 10 and Figure 11 illustrate comparative TF analysis results for the acceleration responses recorded during the slow pedaling trials 1 and 2, respectively. The effectiveness of the TVF EMD is evident in the distinct separation of frequency components. TVF-EMD effectively decomposes the nonstationary acceleration signals into IMFs, isolating modal contributions from the bridge data in the first and second rows. On the other hand, the SET-based results, in Figure 10b and Figure 11b, exhibit reduced frequency resolution and energy concentration, with modal peaks being significantly less distinct. SET is generally considered superior due to its capability to enhance TF concentration by retaining only the most relevant IF trajectories. Unlike the mid-span and quarter-span sensors, which show partially recoverable modal ridges in the first two rows, the IF trajectories extracted from bicycle data in the third row were incoherent and noise-dominated. This outcome contrasts with earlier results in Figure 6 and Figure 7, where the combination of WPT and SET produced well-defined modal ridges under similar excitation conditions.

**Figure 12:**
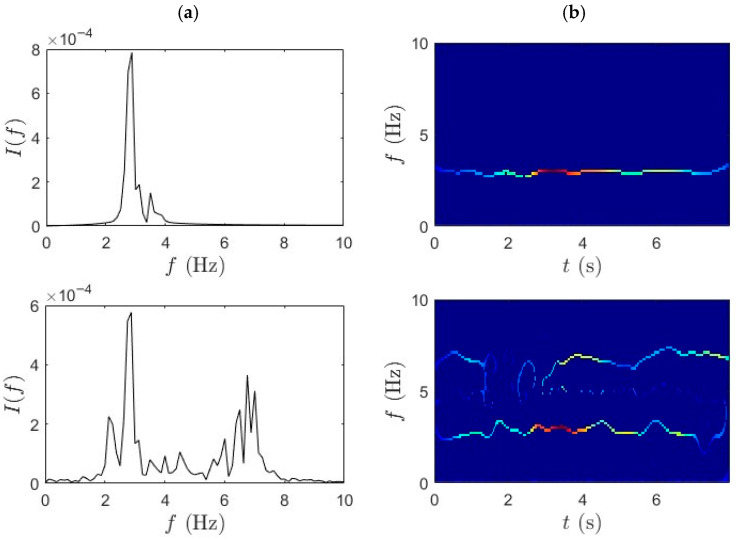

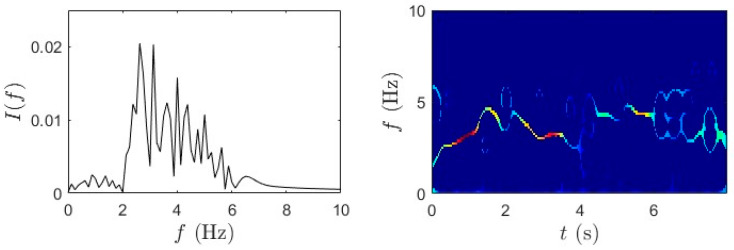
(**a**) TVF-EMD and (**b**) SET results for fast-pedaling trial 1 for bridge mid-span, bridge quarter-span, and bicycle.

**Figure 13:**
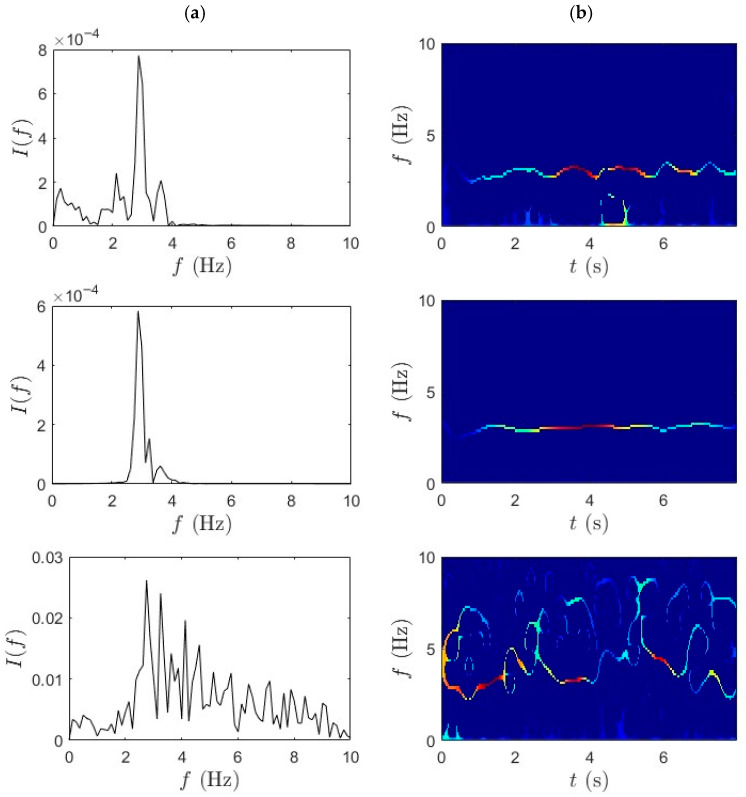
(**a**) TVF-EMD and (**b**) SET results for fast-pedaling trial 2 bridge mid-span, bridge quarter-span, and bicycle.

Similarly, Figure 12 and Figure 13 present the TF analysis of acceleration responses obtained during fast pedaling (high-speed traversal) using TVF-EMD followed by the SET. The performance of TVF-EMD under fast-pedaling excitation was less robust compared to slow-pedaling trials. Specifically, in the third row, corresponding to the bicycle-mounted sensor, TVF-EMD failed to produce clearly distinguishable peaks, resulting in incomplete modal separation. This limitation propagated into the subsequent SET analysis, where the frequency ridges exhibited pronounced fluctuations and instability. The presence of mode mixing in the TVF-EMD output significantly reduced the sharpness of frequency localization and produced blurred and fragmented ridges. By contrast, WPT achieved notably cleaner frequency separation under the same fast-pedaling conditions. WPT demonstrated resilience against mode mixing, maintained well-defined spectral boundaries, and improved decomposition performance. The superiority of WPT in this context suggests that wavelet-based methods are better suited for analyzing short-duration, high-energy excitation events in iBHM.

The qualitative findings are further supported by the quantitative frequency identification results presented in Table 2, which summarizes the modal frequencies extracted using the TVF-EMD method under both slow- and fast-pedaling conditions. During slow pedaling, a consistent frequency of approximately 2.14 Hz was observed at both the mid-span and quarter-span locations. This repeatability suggests that TVF-EMD can capture the fundamental bridge mode under low-energy excitation. In contrast, the bicycle response shows a higher frequency of around 4 Hz. However, in trial 4, the quarter-span data suggests possible mode mixing or spurious component detection. In the fast-pedaling trials, the extracted frequencies increase, reaching up to 3 Hz at mid-span in trial 3. Nonetheless, inconsistencies arise across trials and locations, particularly in the bicycle data, which fluctuates between 2.75 Hz and 3.12 Hz. These variations highlight the limitations in the robustness and repeatability of the TVF-EMD and SET framework under rapidly changing dynamic conditions. The challenges are most evident during fast traversal, where nonstationary signal characteristics and strong coupling effects diminish the accuracy and repeatability of the identified modes. In the third fast-pedaling trial for quarter-span, the signal quality was insufficient to extract modal parameters. The damping ratio values identified using TVF-EMD results are lower for fast-pedaling trials compared to the slow-pedaling trials; however, no clear trend could be identified, which can be attributed to differences in pedaling excitation, short-duration micromobility data, environmental influences, and inherent estimation uncertainties in operational modal analysis.

Although the combination of TVF-EMD and SET provides a baseline capability for frequency identification under dynamic excitation, it does not consistently achieve high resolution or strong separation of modal content in all scenarios. This is especially evident in fast-wheeling cases, where the nonstationary nature of the signal requires more adaptive and energy-focused processing. Quantitative comparison of the frequency estimation accuracy of WPT-SET and TVF-EMD-SET for bicycle data is shown in Table 3. Both methods identified a dominant frequency close to the 2.85 Hz baseline. WPT-SET yields a smaller bias and narrower confidence interval, indicating slightly higher accuracy and repeatability. TVF-EMD exhibited marginally larger RMSE and variance, consistent with its sensitivity to local mode mixing. Frequency shifts can be observed between slow pedaling and fast pedaling in Table 1 and Table 2. At higher pedaling rates, increased rider-induced excitation introduces larger dynamic loads and higher harmonics, which can amplify nonlinear vehicle–bridge interaction effects. This coupling results in slightly elevated identified modal frequencies, as observed in the fast-pedaling trials. The findings confirm that WPT in combination with SET offers a powerful methodology for addressing two common challenges in iBHM: (i) separating closely spaced modal frequencies in nonstationary data and (ii) maintaining spectral clarity under a short observation time and high dynamic loads. It should be noted that, regardless of the signal processing method used, the average results from the fast-pedaling trials align closely with the bridge frequency of 2.85 Hz s (May et al., 2024 [35]). This signifies that pedaling at a higher cadence yields more accurate modal identification for iBHM.

## 5. Conclusions

This study explores the feasibility of using micromobility devices, specifically bicycles, as cost-effective and sustainable platforms for iBHM. The research highlights how easily reliable modal parameters can be extracted utilizing acceleration data without the need for fixed or costly instrumentation. Hence, a promising alternative for BHM with limited accessibility or where permanent monitoring systems may not be practical has been provided. Furthermore, a comparative evaluation of advanced time–frequency signal processing methodologies is conducted, focusing specifically on the combination of WPT–SET, as well as TVF-EMD, with SET. The frequency separation achieved through WPT is notably cleaner and less affected by mode mixing compared to TVF-EMD, indicating enhanced decomposition performance. Notably, the WPT effectively deconstructs nonstationary vibration signals into distinctly separated frequency bands. This decomposition is further enhanced by the SET, which improves the concentration of TF representations, thereby enabling the precise identification of dominant frequencies and damping ratios across varying cycling speeds. In contrast, the TVF-EMD exhibits diminished accuracy, particularly at elevated pedaling rates, where issues such as mode mixing and blurred ridges compromise interpretability. The proposed WPT-SET method focuses on identifying modal parameters using a minimal number of sensors, thereby improving efficiency and reducing costs in the monitoring process. These findings underscore the efficacy of the WPT–SET framework as a sparse yet robust approach for analyzing nonstationary signals, particularly within the context of iBHM applications. Future studies of this topic will focus on the sensitivity analysis of the sensor location on the efficacy of the TF methods analyzed in this study. The dataset used in this study is constrained as it involves a limited number of traversals by a single bicycle under controlled, low-traffic conditions. Factors pertaining to real-world conditions, such as ambient traffic-induced vibrations, varying environmental conditions, and the presence of multiple pedestrians or cyclists, were not investigated in this study. The WPT–SET method maintains stable modal frequency identification across the available traversals; however, it may be challenged under extremely short or high-noise segments, as a formal sensitivity analysis has not been conducted and is reserved for future studies.

## Figures and Tables

**Table 1:** Identified frequencies (Hz) and damping ratio (%) using WPT and SET.

		Mid-Span (Hz)/%	Quarter-Span (Hz)/%	Bicycle (Hz)/%
Slow Pedaling	Trial 1	2.14/3.65	2.14/3.57	4.00/2.32
	Trial 2	2.14/3.75	2.14/3.68	4.00/2.28
	Trial 3	2.16/3.37	2.16/3.46	3.50/3.57
	Trial 4	2.14/2.96	2.14/2.92	4.00/1.65
Fast Pedaling	Trial 1	2.87/3.04	2.87/2.06	2.62/2.07
	Trial 2	2.87/2.47	2.87/2.34	2.75/1.61
	Trial 3	3.00/3.22	3.00/3.22	2.87/2.33

**Table 2:** Identified frequencies (Hz) and damping ratio (%) using TVF-EMD and SET.

		Mid-Span (Hz)/(%)	Quarter-Span (Hz)/(%)	Bicycle (Hz)/(%)
Slow Pedaling	Trial 1	2.14/3.78	2.14/3.78	4.00/3.42
	Trial 2	2.14/3.99	2.14/2.36	4.00/4.61
	Trial 3	2.16/3.33	2.16/4.06	3.50/4.17
	Trial 4	2.14/2.88	3.43/1.19	4.00/2.43
Fast Pedaling	Trial 1	2.87/2.48	2.87/2.83	3.12/2.14
	Trial 2	2.87/2.37	2.87/2.23	2.75/1.57
	Trial 3	3.00/3.15	-	2.87/1.50

**Table 3:** Quantitative comparison of frequency estimation accuracy.

Method	Mean (Hz)	Bias (Hz)	RMSE (Hz)	Std. Dev (Hz)	95% CI (Hz)
WPT-SET	2.75	−0.10	0.10	0.13	2.59–2.91
TVF-EMD-SET	2.91	0.06	0.13	0.19	2.99–3.16

## Data Availability

The data that support the findings of this study are available from the benchmark data source of the cited reference [35].
